# Analysing the quality of Swiss National Forest Inventory measurements of woody species richness

**DOI:** 10.1186/s40663-020-00252-1

**Published:** 2020-06-17

**Authors:** Berthold Traub, Rafael O. Wüest

**Affiliations:** 1 Scientific Service NFI, Swiss Federal Research Institute WSL, Zürcherstrasse 111, 8903 Birmensdorf, Switzerland; 2 Spatial Evolutionary Ecology, Swiss Federal Research Institute WSL, Zürcherstrasse 111, 8903 Birmensdorf, Switzerland

**Keywords:** Biodiversity, Data quality, Equivalence test, Forest inventory, Monitoring, Observer agreement, Richness, Pseudo-turnover

## Abstract

**Background:**

Under ongoing climate and land-use change, biodiversity is continuously decreasing and monitoring biodiversity is becoming increasingly important. National Forest Inventory (NFI) programmes provide valuable time-series data on biodiversity and thus contribute to assessments of the state and trends in biodiversity, as well as ecosystem functioning. Data quality in this context is of paramount relevance, particularly for ensuring a meaningful interpretation of changes. The Swiss NFI revisits about 8%–10% of its sample plots regularly in repeat surveys to supervise the quality of fieldwork.

**Methods:**

We analysed the relevance of observer bias with equivalence tests, examined data quality objectives defined by the Swiss NFI instructors, and calculated the pseudo-turnover (PT) of species composition, that is, the percentage of species not observed by both teams. Three attributes of woody species richness from the latest Swiss NFI cycles (3 and 4) were analysed: occurrence of small tree and shrub species (1) on the sample plot and (2) at the forest edge, and (3) main shrub and trees species in the upper storey.

**Results:**

We found equivalent results between regular and repeat surveys for all attributes. Data quality, however, was significantly below expectations in all cases, that is, as much as 20%–30% below the expected data quality limit of 70%–80% (proportion of observations that should not deviate from a predefined threshold). PT values were about 10%–20%, and the PT of two out of three attributes decreased significantly in NFI4. This type of uncertainty – typically caused by a mixture of overlooking and misidentifying species – should be considered carefully when interpreting change figures on species richness estimates from NFI data.

**Conclusions:**

Our results provide important information on the data quality achieved in Swiss NFIs in terms of the reproducibility of the collected data. The three applied approaches proved to be effective for evaluating the quality of plot-level species richness and composition data in forest inventories and other biodiversity monitoring programmes. As such, they could also be recommended for assessing the quality of biodiversity indices derived from monitoring data.

## Background

Biodiversity is important for sustaining ecosystem functioning (Tilman et al. 2014) but can also constitute, promote and stabilize ecosystem services (Balvanera et al. 2006; Cardinale et al. 2012; Mace et al. 2012). Under ongoing climate and land-use change, biodiversity is continuously decreasing, which in turn threatens nature’s contribution to human livelihood and well-being (IPBES 2018). Therefore, monitoring biodiversity is a valuable and feasible approach to assess the state and trends of ecosystem functioning and services. National Forest Inventories (NFIs) were initially set up to provide statistically reliable estimates of timber-related resources to stakeholders such as politicians, ecologists, forest services and the timber industry, and to national and international organizations and projects. Since the importance and demand for quantitative information on aspects of biodiversity are growing, NFIs have gradually included attributes of structural diversity (Storch et al. 2018; Brändli and Hägeli 2019), species richness and species composition, which are highly relevant for reporting biodiversity indicators (FOREST EUROPE 2015). Their long history (Norway’s NFI just celebrated its 100th birthday with a conference; NIBIO 2019) means that they have produced long-term data series on biodiversity. These time series can be used to assess the effect of past changes or the success of mitigation measures on biodiversity and ecosystem services. However, robust assessments of changes in monitoring or survey data depend on high-quality data.

Collecting data for biodiversity monitoring in general, and forest inventories in particular, usually involves resource-intensive fieldwork on a large number of sample plots. Most of the recorded data are, however, expert judgements (e.g. on forest structure or the identity of species) rather than measurements (e.g. tree diameter or height). Assessing the quality of recorded biodiversity indicators hence essentially translates into quantifying observer error typically associated with overlooking or misclassifying species. Observer error is comprehensively investigated in forest health monitoring programmes (e.g. Allegrini et al. 2009; Bussotti et al. 2009; Ferretti et al. 2014) and in vegetation surveys (e.g. Vittoz et al. 2010; Burg et al. 2015; Morrison 2016). Observer agreement, the inverse of observer error, refers to the extent of agreement between observer ratings, quantified by measures such as agreement coefficients (Gwet 2012). Most studies on the quality of vegetation surveys use a predefined experimental design to evaluate the reliability of results by assessing the level of agreement between many observers that record biodiversity in the same plots. Quality assessment and control frameworks, as established in NFIs, usually evaluate data quality based on repeat or control surveys, where 5%–10% of all plots are revisited by different (groups of) observers (Tomppo et al. 2010). These surveys focus on the evaluation of data quality in terms of the reproducibility of the assessments, determined by the variation in measurements made on a subject under changing conditions, e.g. due to measurements being made by different observers (Bartlett and Frost 2008).

We analysed the quality of woody species richness data assessed in the Swiss NFI and addressed the questions: (i) Is the detected magnitude of observer bias relevant? (ii) Does data quality meet expectations defined by data quality objectives? (iii) Has the quality of species identification in the Swiss NFI improved over time? In the following, we provide an overview of the approaches used to address these questions and how they are best applied for data collected from Swiss NFI repeat survey data. Finally, we discuss how the answers to these questions can help improve data quality in vegetation surveys in general and in NFIs in particular.

## Methods

### Data sources

The Swiss NFI is a multisource and multipurpose forest inventory. The field measurements encompass about 6400 permanent sample plots, arranged on a systematic 1.4 km × 1.4 km sampling grid. Each sample plot consists of two concentric circles of 200 m^2^ and 500 m^2^ and an interpretation area of 50 m × 50 m. Starting with NFI4 (2009–2017), continuous fieldwork has been carried out over a nine-year inventory cycle. Each annual survey (panel) is representative of the entire country and covers one-ninth (about 700 plots) of the complete sample. In total about 280 attributes are assessed per sample plot; many of them cover tree and stand characteristics, but several attributes concern species richness. Details on the methods and the design of the Swiss NFI are presented in Fischer and Traub (2019). In the NFI4 a total of twenty employees were hired, but the majority of fieldwork was conducted by four teams of two employees, who assessed about half of the sample plots (2748 out of 5641). In NFI3 (2004–2006) forty employees were hired, and about half (3313 out of 6914) of the sample plots were visited by eight teams.

The annual repeat surveys are a pillar of the quality assessment and control framework of the Swiss NFI (Traub et al. 2019). Since the first NFI (1982–1986), they have been carried out on a varying random subsample of the NFI panel to evaluate the reproducibility of survey measurements. The repeat surveys are carried out by the field teams in parallel to the fieldwork of the regular annual surveys. The allocation of teams to the repeat survey is solely driven by organizational aspects and by the rule, that teams never revisit their own plots (Cioldi and Keller 2019). About 9% (626) of the sample plots in NFI3 and 8% (438) in NFI4 were revisited with a repeat survey. The majority of the field work was conducted by seven teams in NFI3 and four teams in NFI4 who managed about the half of the repeat survey. All attributes of a plot are remeasured using the same methods and equipment as for the regular survey. The data assessed by the regular field team are not accessible during the repeat surveys to assure an independent re-measurement of the plot (‘blind check’). With this type of repeat survey nothing can be said about the correctness of the results stemming from either the regular or the repeat survey, since the true attribute value is unknown. That is, the validity of the results or any attribution of performance to individual teams cannot be derived, and consequently observer error cannot be ascertained. During the entire field season (April–November), a team manages to assess two sample plots per day on average, and thus the resources needed for the repeat survey can roughly be derived from the number of repeat survey plots assessed.

We analysed NFI3 and NFI4 data and investigated the reproducibility of three attributes that are basic elements of biodiversity indicators: (i) occurrence of tree and shrub species that reach 40 cm in height but are less than 12 cm in diameter at breast height, assessed on the 200 m^2^ circle of the concentric NFI sample plots (WoodySp); (ii) number of tree and shrub species at the forest edge, assessed along a line up to 50 m in length (FoEdge); and (iii) main shrub and trees species in the upper storey of the relevant stand with crown cover ≥5%, assessed on the 50 m × 50 m interpretation area (UpStorey). The attribute UpStorey corresponds to Indicator 4.1 ‘tree species composition’ of Forest Europe (FOREST EUROPE 2015). All species were selected from the exhaustive species list of woody plants, as defined in the NFI field survey manual (Düggelin 2019). In Table 1 general statistics of the examined attributes are presented.

**Table 1:** Statistics of richness for the three attributes woody species (WoodySp), forest edge species (FoEdge) and upper storey species (UpStorey). Cv (%): coefficient of variation; Med: median. Database: regular field survey of NFI3 and NFI4 (accessible forest without shrub forest)

NFI						Repeat survey
	Cycle	Mean	Ccv (%)	Med	***n***	***n***
WoodySp	4	9.24	71.53	8	5641	438
	3	7.85	72.44	6	6914	626
FoEdge	4	11.87	41.86	12	831	60
	3	9.84	47.31	10	1010	95
UpStorey	4	2.89	52.85	3	3874	268
	3	2.70	52.87	3	5079	398

**Fig. 1:**
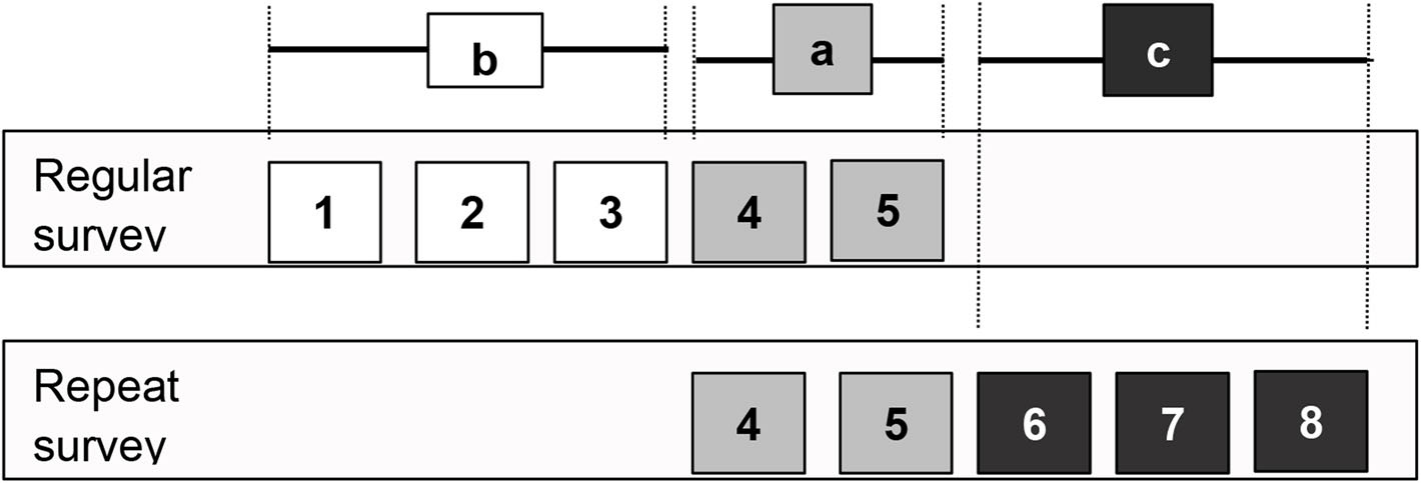
Schematic representation of the components of species richness assessment and pseudo-turnover (PT) situations, adapted from Baselga (2012). Each number represents a different species. a: number of species reported in both surveys; b: number of species reported only in the regular survey; c: number of species reported only in the repeat survey; a + b: richness of regular survey; a + c: richness of repeat survey

A schematic representation of the components of richness assessments is illustrated in Fig. 1, where (a) denotes the number of species present on both occasions, (b) the number of species reported in the regular survey but not in the repeat survey, and (c) the number of species reported in the repeat survey but not in the regular survey (Baselga 2012). Nestedness is a special situation where the composition of species observed on one occasion is a subset of the composition of species recorded on the other occasion. In this case either (b) or (c) equals zero (but not both).

### Magnitude of observer bias

From a statistical point of view, the richness data of the regular and the repeat survey were collected from a paired sample, assuming that the observed subjects and their true attribute values remained unchanged between the regular and repeat survey. Observed differences are usually evaluated by hypothesis tests, such as a paired t-test, or by visual inspection of the corresponding confidence intervals (e.g. Kercher et al. 2003; Burg et al. 2015; Traub et al. 2019). However, the aim in analysing observer bias is to demonstrate that the mean difference in richness is zero. In a classical t-test, with ${H}_0:\overline{x}-\mu =0$ and ${H}_A:\overline{x}-\mu \ne 0$, an effect such as a bias can be demonstrated if *H*_0_ is rejected. However, one cannot conclude that there is no bias if *H*_0_ is not rejected by this type of hypothesis specification. Consequently, in our study the agreement or equivalence between richness measurements of regular and repeat surveys was evaluated by the ‘two one-sided t-test’ (TOST) method for paired samples, a standard test of equivalence. Basic assumptions of the TOST method can be found in Schuirmann (1987), and a general overview and discussion of equivalence tests is provided by Walker and Nowacki (2011), Mara and Cribbie (2012), Ialongo (2017) and Lakens (2017). An application of equivalence tests in forestry in the context of modelling is found in Robinson et al. (2005). The formal notation of TOST hypotheses was: *H*_0_ : *μ* ≤ *μ*_0_ – *m* or *μ* ≥ *μ*_0_ + *m* and *H*_*A*_ : *μ*_0_ – *m* ≤ *μ* ≤ *μ*_0_ + *m*. The target variable *μ* was derived from the richness difference between the regular and repeat survey, with *μ* = (*a* + *c*) − (*a* + b) = *c* − *b* (Fig. 1), *m* = critical margins of relevance (a subjective choice of an interval within which richness differences are considered negligible or not relevant), and *μ*_0_ = 0 (the value *μ* is tested against). The calculation of t-values is explained in Additional file 1: Eq. S1.

*H*_0_ of the TOSTs stated that the mean richness would differ by more than the critical margins. If *H*_0_ was rejected_,_ we concluded that the results of the regular and the repeat survey were equivalent for practical purposes, that is, observer identity had no practical impact on the mean richness values from the survey sample. The critical margins *m* were determined by the NFI instructors based on their expectation of richness differences that should not be exceeded. They decided on a maximum difference of ± 2, 3 and 0 species as critical margins for the attributes WoodySp, FoEdge and UpStorey, respectively. Following the central limit theorem, we assumed that the mean of richness differences was normally distributed and that the sample size for all attributes was always *n* ≥ 60. We evaluated the results by interpreting confidence intervals (CIs) to conclude if richness measurements were equivalent. Details on the construction and interpretation of TOST-based CIs are given in Additional file 1: Figure S1. The TOSTs were completed with SAS PROC T-TEST (SAS Institute 2014). Details on the computational methods are presented in SAS Institute (2013).

### Assessing data quality objectives

The assessment of observer bias, based on the deviation in reported species richness between observers, already delivers valuable information about data quality. Equal richness values, however, could be obtained from completely different species compositions resulting from high rates of misidentification, which is not in harmony with the goal of achieving the highest possible data quality. The data quality objectives (DQO) method involves a more detailed evaluation of the variability between the regular and repeat surveys; as explained above, even in the absence of bias, results may still lack sufficient observer agreement in terms of precision.

DQOs quantify the degree to which we are willing to accept this deviation between observers by applying: (i) quantifiable threshold values, called measurement quality objectives (MQOs), which define a tolerance level of the sum of exclusive species (b + c) which should not be exceeded; and (ii) data quality limits (DQLs), which define the proportion of measurements expected to comply with the MQOs (Allegrini et al. 2009; Ferretti 2009). Data quality results (DQR) constitute the observed proportion of cases compliant with the MQO, i.e. the proportion of measurements that do not exceed the MQO. The DQOs of the examined attributes are listed in Table 2. The DQO narrative for the example of WoodySp would read: “The sum of exclusive species must not exceed two species and we expect this limit to be met in at least 80% of all observations.” The MQOs and DQLs defined by the NFI instructors were based on their best guess of what experienced field teams should be able to achieve in the long term, rather than optimal results under ideal conditions (Pollard et al. 2006). At the same time, the MQOs reflect the degree of deviation that is thought to be non-trivial or practically important.

**Table 2:** DQO specification for the sum of exclusive species. MQO: measurement quality objectives (tolerated sum of exclusive species b + c, DQL: data quality limits (expected proportion of samples that meet the MQO)

Attribute	MQO (b + c)	DQL (%)	Coverage threshold^a^
WoodySp	2	80	< 5%
FoEdge	3	80	
UpStorey	0	75	< 6%

^a^Coverage threshold: exclusive species with coverage lower than the threshold were not counted

The DQO definition can easily be transformed into a one-sided test for binomial proportions (Zar 2010) that assesses whether the actual DQR proportion ${\hat{p}}_{DQR}$ significantly exceeds the expected DQL proportion *p*_*DQL*_ based on the hypotheses *H*_0_ : *p*_*DQR*_ ≤ *p*_*DQL*_ (the DQR is inferior to the DQL) and *H*_*A*_ : *p*_*DQR*_ > *p*_*DQL*_ (the DQR is superior to the DQL – which would demonstrate sufficient data quality). The DQR proportion is calculated as ${p}_{DQR}=\frac{1}{n}\sum \left({MQO}_{compl}\right),$ where *MQO*_*compl*_ is calculated per sample plot by *MQO*_*compl*_ = 1 *if* (*b* + *c*) ≤ *MQO* and 0 otherwise. The binomial test statistic is computed as: *z* = (*p*_*DQR*_ − *p*_*DQL*_)/*stderr*_*DQL*_, with ${stderr}_{DQL}=\sqrt{p_{DQl}\left(1-{p}_{DQL}\right)/n}$ (Zar 2010, eq. 24.30). By constructing a confidence interval of *p*_*DQR*_, we can evaluate *H*_*A*_ : *p*_*DQR*_ > *p*_*DQL*_. Several methods were applied to calculate CIs for the binomial proportions, but their differences were found to be marginal. For the interpretation of results, we used the ‘Wilson’ CI because of its good performance, as stated by Newcombe (1998) and SAS Institute (2014). Inferiority is found if the upper limit of the CI is below the DQL and superiority if the lower limit of the CI is above the DQL. If the upper limit of the CI exceeds the DQL, results are deemed inconclusive (non-inferiority is not impossible but also not significant). According to Cochran (1977), the relationship between sample size and the width of a confidence interval of a binomial proportion (such as the DQR) can be estimated on the basis of the normal approximation for infinite populations by $n\approx \frac{t_{\alpha /2}^2\ p\left(1-p\right)\ }{d^2}$, where *d* = half the width of the 1– *α* confidence interval calculated by $d={t}_{\alpha /2}\sqrt{\frac{p\left(1-p\right)}{n}}$, with *p* = *p*_*DQR*_. All CIs and binomial tests were calculated with SAS PROC FREQ (SAS Institute 2014).

### Pseudo-turnover and quality development

So far, we have focused on richness differences and on the number of exclusively found species as measures of observer bias and uncertainty. As an additional analysis, turnover assessment involves investigating the agreement in species identifications between observers, thus providing a more differentiated picture of data quality. Here we used pseudo-turnover of species composition as defined by Nilsson and Nilsson (1985), but we acknowledge that any of the available turnover measures could have been used instead (cf. Tuomisto 2010 for an extensive overview). Pseudo-turnover is defined as *PT* = (*A* + *B*)/(*S* _ *A* + *S* _ *B* ) × 100, where *A* and *B* represent the number of species exclusively found by team A/B, and the terms *S* _ A and *S* _ *B* denote the sum of all species found by team A/B (*α* diversity of team A and team B). According to the notation of Baselga (2012), PT can equivalently be expressed as *PT* = (*b* + *c*)/(2*a* + *b* + *c*) × 100 (Fig. 1). PT is widely used when assessing reproducibility in vegetation surveys, where values are typically 10%–30% (Morrison 2016).

The definition of Nilsson’s pseudo-turnover (PT) enables the direct and simple interpretation of results as the proportion of disagreement. For example, an inter-observer PT of 30% indicates that 30% of species reported were not observed by both teams (Morrison 2016). Thus, PT is an indispensable component in the evaluation of the data quality of a species diversity assessment.

The attempt to define DQOs for PT led to ambiguous results and was not applied in this study. We have focused instead on the development of PT between inventory cycles, which provides a useful instrument to judge the development of observer agreement. For the construction of CIs, we used the ratio of mean estimator (Cochran 1977). Details on the CI construction are provided in Additional file 1: Figure S4.

### Power analysis

A power analysis may reveal whether remeasuring 8%–10% of the plots is sufficient to detect relevant effects. Power is the probability of rejecting the null hypothesis when the alternative is true, that is, the probability of rejecting a false *H*_0_. Given alpha and *n*, a certain non-trivial effect (e.g. the difference between population means) can be detected with a certain power. The larger the effect, the more power. The power in an equivalence test on richness difference is the probability of rejecting non-equivalence when the richness assessment in fact is equivalent, that is, the probability of observing the mean difference within the margins when the true value lies within the margins. The DQO power analysis corresponds to the z-test for binomial proportions. The power analysis of PT values (detection of change between NFI cycles) is based on the ‘two sample t-test for mean differences with unequal variances’. All power curves were created with SAS PROC POWER (SAS Institute 2014).

## Results

### Magnitude of observer bias

**Fig. 2:**
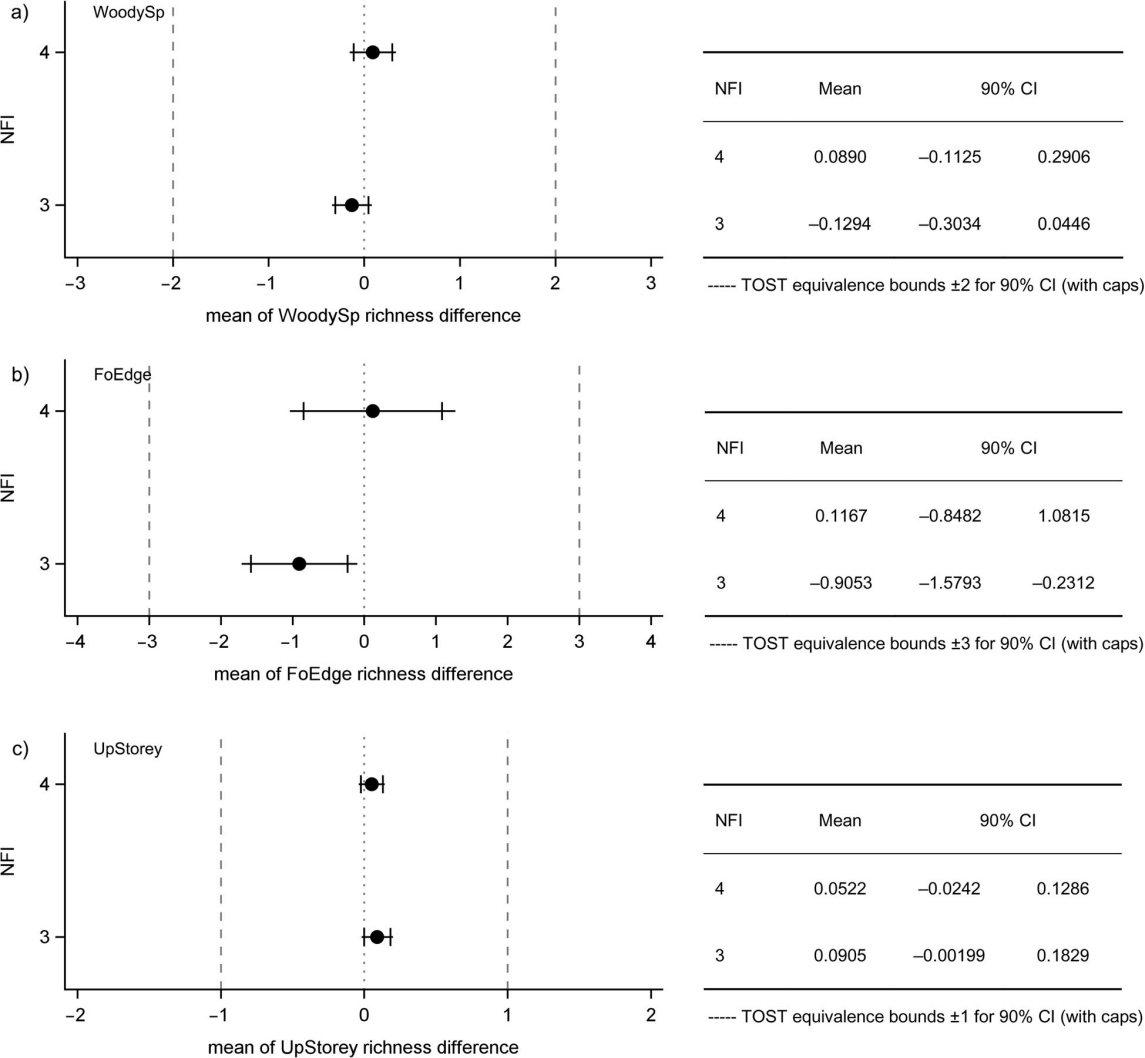
Results from equivalence tests comparing NFI3 and NFI4 richness differences in: (**a**) woody species (WoodySp), (**b**) forest edge species (FoEdge), and (**c**) upper storey species (UpStorey). The horizontal lines are the confidence intervals (dot = mean). The caps mark the 90% CIs and the ends of the horizontal lines mark the limits of the 95% CIs. The dashed, vertical lines are the margins within which results were considered equivalent. Exact values of the means and 90% CIs are given to the right of the figure for NFI3 and NFI4

Equivalence of richness difference could be demonstrated for all attributes in both NFI cycles. The 90% CIs (horizontal line with cap) were entirely contained within the equivalence margins (Fig. 2); the confidence limits were substantially far from the specified margins that indicate the threshold to relevant bias, even in the case of attribute FoEdge with a large CI. All *p*-values of the corresponding TOSTs were <0.0001 and thus the *H*_0_ were rejected. We conclude that no significant bias exists for any attribute. The 95% CI (horizontal line without cap) indicates whether a classical t-test would assume significant bias if these intervals do not overlap with zero. Our results show that the t-test would indicate significant bias for the FoEdge attribute in the NFI3 data (mean = − 0.91, *t* = − 2.23, *p* > |*t*| = 0.028).

With regards to sample size and power analysis, we analysed the NFI4 data of the attribute WoodySp as an example. Based on the stddev of 2.56, a sample size of about 42 observations is sufficient to reach a power of 80% given a mean expected richness difference (effect) of ±1. As the effect approaches the critical margin of ±2, more observations are needed to gain this power (*n* = 164 and *n* = 450 for effects of 1.5 and 1.7, respectively). The power as a function of sample size and effect size is presented in Additional file 1: Figure S2.

### Assessing data quality objectives

**Fig. 3:**
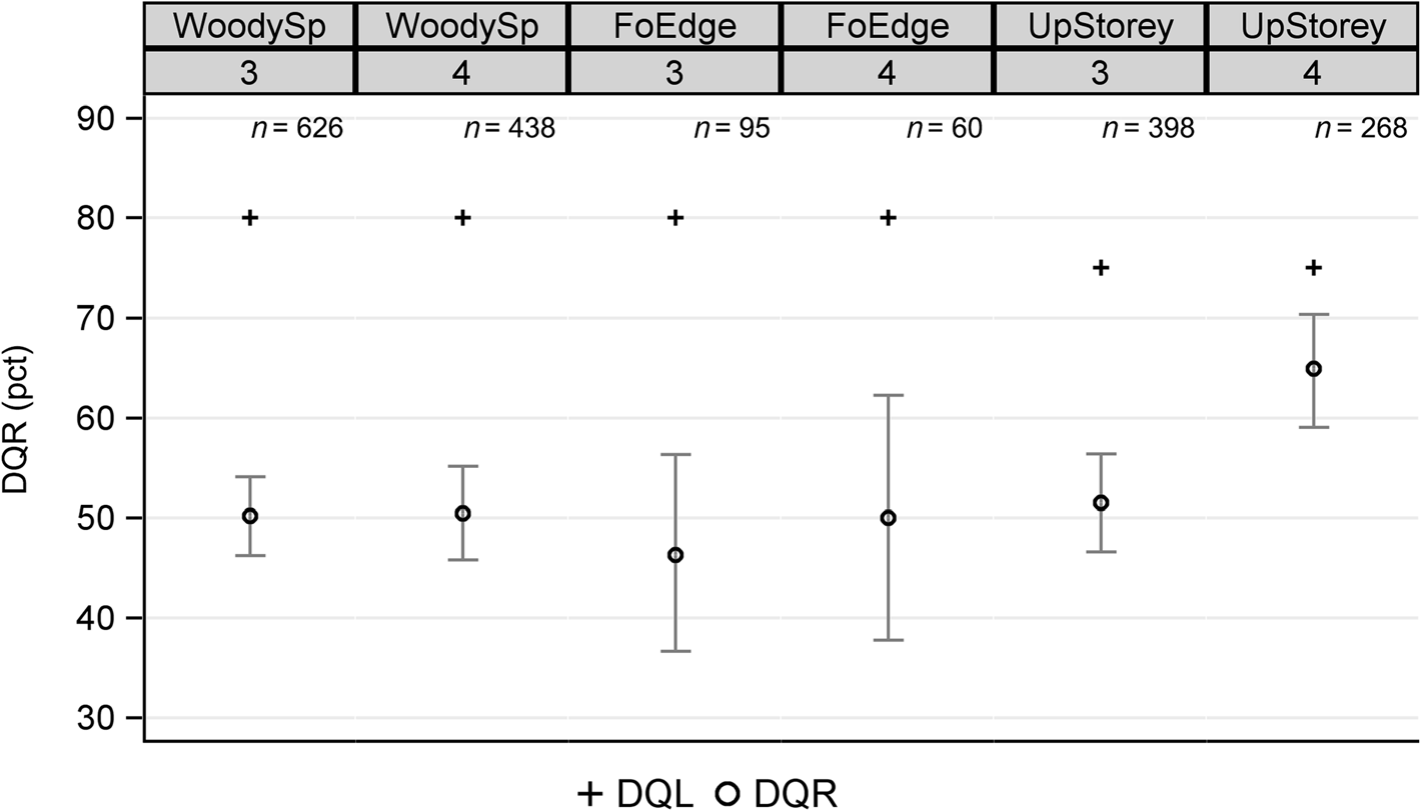
DQR results of the NFI3 and NFI4 repeat surveys for woody species (WoodySp), forest edge species (FoEdge) and upper storey species (UpStorey). Crosses depict data quality limits (DQL) and circles refer to the data quality results (DQR), i.e. the observed mean results comparing regular and repeat surveys with their 95% confidence intervals (Wilson)

The results of the DQO analysis revealed that the data quality for all examined attributes in both inventory cycles was below the expectations of the NFI instructors (Fig. 3). The upper limits of the CIs were all below the DQL, indicating that the quality in richness assessments was substantially inferior to the objectives expressed as DQLs. The percentage of nested sample plots, an indicator of the proportion of overlooked species, varied between 24.25% (UpStorey NFI4) and 38.95% (FoEdge NFI3). Only the quality of the attribute UpStorey improved substantially in NFI4; the DQR increased from 51.51% to 64.93% and the nestedness decreased from 39.20% to 24.25%. The complete results are given in Additional file 1: Table S1.

Since the CIs of the DQR do not encompass the specified DQL, no effect exists in terms of the specified ${H}_A:{\hat{p}}_{DQR}>{p}_{DQL},$ and in that sense power and sample size calculations have no meaning. Nevertheless, we carried out a power analysis using hypothetical effect sizes. Under *H*_0_ = 0.8, the analysis showed that a sample size of 368 plots is needed to detect an effect of 0.05 (that is, a DQR proportion of 0.85) with a power of 80%. A sample size of at least 498 plots is needed to detect this effect with a power of 90%. More details on sample size and power for four examples of DQR proportions can be found in Additional file 1: Figure S3.

### Pseudo-turnover and quality development

**Fig. 4:**
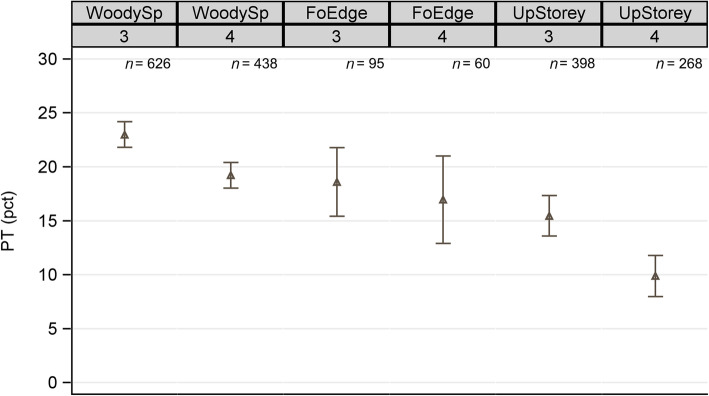
Mean and CIs of pseudo-turnover (PT) for the three assessed richness attributes woody species (WoodySp), forest edge species (FoEdge) and upper storey species (UpStorey) in NFI3 and NFI4. The mean (triangles) and stderr of the CI (grey whiskers) were calculated using the ratio of means estimator

Pseudo-turnover (PT) between the regular field survey and the repeat survey ranged from 15.45% to 22.98% in NFI3 and from 9.88% to 19.22% in NFI4 (Fig. 4). In both NFIs, we observed the highest PT for woody species composition and the lowest for upper storey species, with intermediate values for forest edge species composition. We found generally lower PT in NFI4 compared with in NFI3, with significant differences for upper storey and woody species composition (non-overlapping CIs in Fig. 4), but a non-significant decrease in PT in forest edge composition. Detailed results of the PT analysis are presented in Additional file 1: Tables S2 and S3; Figure S4 shows that estimating the PT CIs in different ways would not have changed the interpretation of results.

The analysis of the PT components (Additional file 1: Table S3) revealed different reasons for the observed changes in PT. Whereas the improvement in attribute WoodySp was largely based on a significant increase in the number of species found in both surveys (a) from 6.40 (*stderr* = 0.18) in NFI3 to 7.86 (*stderr* = 0.27) in NFI4, the number of exclusive species (b + c) was not significantly lower in NFI4. Contrarily, the improvement in PT for the attribute UpStorey was predominantly caused by a significant decrease in the number of exclusive species from 0.86 (*stderr* = 0.06) in NFI3 to 0.61 (*stderr* = 0.06) in NFI4. The PT value and its components of the FoEdge attribute showed a neutral behaviour: both exclusive species and those reported in both surveys have not significantly changed.

The power analysis was calculated for the attribute WoodySp as an example, based on a standard deviation of 15% in NFI3 and 12% in NFI4, with a sample size relationship of 3/2. It revealed that a power of 80% could be reached for a PT difference of 5%, 4% and 3% with at least 235, 365 and 645 observations, respectively. A graphical representation of the relationship between sample size and power to detect significant PT differences is presented in Additional file 1: Figure S5.

## Discussion

Our analyses show in general that the quality in species assessments has increased from the third to the fourth NFI cycle. We could further demonstrate equivalence in richness assessment, the pre-defined data quality objectives, however, have not been met. Our study also shows that species turnover has decreased from NFI3 to NFI4. Below, we discuss the three investigated research questions, critically examine statistical aspects of our approaches, and discuss implications and potential extensions of our work. Although biodiversity monitoring and inventories vary in many aspects – measurement protocols, time available for the assessment, and the level of training to name just a few – we also compare our findings with data quality assessments of diversity indicators from other inventories wherever possible.

### Interpretation of observer bias

Since we expect observers to come to the same result when assessing richness attributes on unchanged NFI sample plots, we hope to find evidence for equivalence rather than difference in the richness value. The results of the applied TOST equivalence tests consistently demonstrated equivalence, that is, we found no systematic deviation (bias) for any attribute examined in both NFI cycles. In other words, the Swiss NFI would not need to worry about data quality if reporting species richness were the sole relevant indicator. We also observed that the classical t-test on differences indicated significant bias in one case, a discrepancy that highlights that using t-tests is problematic when aiming to prove that richness differences do not significantly deviate from zero. Since the probability of rejecting the null hypothesis increases as sample size increases, the TOST approach is more robust in that the conclusion of equivalence does not change with increased sample size. A detailed discussion on this issue is found in Mara and Cribbie (2012).

The definition of critical margins in TOST introduces an additional element into the testing method, but any serious planning of an experiment based on sample size and power calculations requires that one defines the practical relevance of an effect as well.

### Assessing data quality objectives

The DQR proportions and associated CIs from the repeat survey samples revealed that the data quality objectives in terms of species richness are currently not met in the Swiss NFI. The gap of up to 30% to the target objectives is certainly large. Several aspects could contribute to this result. The instructors could simply have overestimated the performance of the observers: were the MQOs set unrealistically low, or the DQL unachievably high? We observed that the instructors managed to define the MQO quickly and with reasonable confidence, whereas the expected DQL proportion was thoroughly debated, which could suggest that DQLs were set at rather large values. On the other hand, the instructors ended up using the DQL as an important MQO-‘waiver’ that enabled them to cope with the difficulties in richness assessments, bearing in mind (i) the demanding situations during field measurement and (ii) the general performance of survey teams perceived during the past field seasons, which should prevent overly high expectations in terms of DQL. The data at hand does not provide a definitive answer, suggesting that targeted tests that determine the accuracy of re-surveys by a single person might be needed. Knowing how well an observer can replicate its own assessment should help determine realistic MQOs and DQLs.

The results, however, could also reflect that training for the observers is simply insufficient or inadequate. Discussing this aspect at length goes beyond the scope of this study and demands a critical and thorough inspection of the training activities within the Swiss NFI.

Although we could not find a conclusive explanation for the failed DQO tests, the potential for data quality monitoring using this method is clear. DQOs are applied in different fields of quality assessments in forest monitoring. Allegrini et al. (2009) applied DQO in the context of ICP-Forests quality assurance procedures, and Bussotti et al. (2009) applied DQO to monitoring tree crown conditions. A comprehensive set of DQO definitions and results is available for the US forest inventory FIA (Pollard et al. 2006). Gasparini et al. (2009) assessed the quality of photo interpretation as applied in the Italian NFI. For the evaluation of the control survey data of the Japanese NFI, MQOs for tree species richness were defined in terms of a coefficient of variation (cv) threshold (Kitahara et al. 2009), which makes sense if a reference value (control group) exists. This type of DQO, however, is not applicable to the Swiss NFI, since a true reference value is not available from the repeat survey.

### Pseudo-turnover and quality development

The pseudo-turnover assessment demonstrated that the quality in species determination increased significantly from NFI3 to NFI4. The three investigated attributes differed with respect to data quality as assessed by PT, but compared quite well with values known from the literature. Our average results from the latest inventory cycle 2008–2017 (NFI4) of woody species in the 200 m^2^ circle (WoodySp, *PT* = 19.22, *stderr* = 0.6) and along the forest edge (FoEdge, *PT* = 16.95, *stderr* = 2.03) were quite close to results published for the Japanese NFI (17.3%, Kitahara et al. 2009), while PT values of tree species in the upper storey (UpStorey, *PT* = 9.88, *stderr* = 0.97) were even better. The lowest PT for UpStorey richness and highest value for WoodySp richness could be explained by the fact that UpStorey species richness on average is much lower than WoodySp, which lists all woody species in the more diverse understorey, because the variance increases with the mean. However, the average of species richness is only one of several determinants of quality and PT. For example, FoEdge richness should in theory show the highest PT values because it has the highest average richness, but it actually exhibits intermediate values. Other factors related to the complexity in recording (such as correctly setting the start and end points of the transect, which determines which individual trees belong to the forest edge) could be linked to the small and insignificant quality increase in forest edge richness in NFI4. The power analyses indicate that repeating ca. 10% of all survey plots provides sample sizes (up to approx. 440 in NFI4) that are large enough to achieve decent power. This is congruent with the recommendation of several authors and customary practice in NFIs. Optimization towards the minimal required fraction of repeated plots, however, requires in-depth power analyses.

### Implications for the Swiss NFI

As a first implication of this study, the data quality of all investigated richness attributes improved from NFI3 to NFI4. This increase in quality can be expected because knowledge, as well as the amount of advanced training of the observers, has steadily increased over time. Moreover, data for NFI4 was recorded continuously by a core of four teams over the period of 9 years, whereas the data for NFI3 was collected within a period of 3 years by a core of seven teams. Hence, switching the data collection system can be considered a good choice in terms of the quality of biodiversity data.

A second implication relates to how data quality can be further improved. Differences in species richness and pseudo-turnover between regular and repeat surveys are mainly caused by two factors: misidentification and overlooking of species. For example, Archaux et al. (2009) reported that on average 15.5% of shrubs and trees taller than 2 m were overlooked and 2.3% were misidentified in his analysis of French ICP-level II plots. Misidentification can be prevented by improved training. However, additional training comes with additional costs, and it is of crucial importance that the resources required for additional training are viewed in relation to the expected benefit. A decision on the amount and form of additional training must therefore involve not only the instructors and field observers, but also stakeholders such as funding agencies. A rigorous and diversified data-quality assessment, such as the one presented in our study, will provide extremely useful information about expectations and can help in reaching such a decision.

The issue of overlooking unfortunately cannot be eliminated by improved training, but requires a larger sampling effort either by spending more time on a plot or by adding more observers. However, even though a greater sampling effort decreases error from overlooking and could also reduce misidentification, organizational constraints and budget limitations render additional sampling effort unfeasible in the Swiss NFI – a situation that is likely paralleled in other inventories and biodiversity surveys. The current Swiss NFI standard of working as teams already appears to be a good measure to overcome overlooking, given that Vittoz and Guisan (2007) found that pairs of observers overlook 10%–20% fewer species than single observers.

We further emphasize the need for additional research because our analyses do not answer all questions related to data quality. Apart from the additional research that we mentioned when discussing the specific research questions, we propose the following avenues of future research. First, we suggest an in-depth analysis of the effect of overlooking species on data quality. The nestedness component in turnover analyses (Baselga 2010), that is, cases where the species composition of one survey is a subset of the composition recorded in the other survey, should provide insight into overlooking error, as it is the main cause of nestedness. Second, we suggest identifying sets of species where observers frequently disagree. We suspect that closely related species that are difficult to distinguish (e.g. within the genera *Tilia* or *Quercus*) might contribute to pseudo-turnover to a greater extent than species that are easier to distinguish. Third, we imagine that small, targeted experiments could help answer open questions. For example, experiments where observers and/or field teams have to identify species in standardized (or artificially created) plots that harbour various combinations of species in a fully-crossed experimental design would not only shed light on intra-observer agreement but would also make it possible to properly assess bias with a given standard. Fourth, the repeat survey approach itself is not optimal to examine the underlying causes of deviations between regular and repeat surveys. We do not know the true richness values in the NFI sample plots because there is no constant control, in other words, no instructor team that does all repeat surveys and as such can serve as the reference against which deviations can be compared. Moreover, the observer combinations (the composition of field teams in the regular survey and the repeat survey) are assigned randomly to the sample plots, which makes it difficult to identify observer combinations that have substantially larger mean richness deviations compared with others. Analysing control survey data could investigate the impact of individual survey team members by investigating the variation in richness differences using multiple-membership models.

## Conclusions

With respect to our specific study system, we conclude that the Swiss NFI needs to decide if additional training for the field crew is needed or if adjusting the quality objectives is necessary to reach the currently unmet data quality objectives in the future. Our results may not produce sufficient insight to reach a conclusion regarding this question, but they certainly provide guidance for identifying additional investigations. Such studies should include targeted, small-scale experiments. In combination with control surveys that set the standard against which repeat survey results can be compared, these experiments will make it possible to determine realistic quality objectives.

More generally, and of importance to any inventory or monitoring programme that surveys species richness, the combination of the three approaches used in this study provides a multi-faceted assessment of data quality. Furthermore, we emphasize that statistical rigour is the only way to prevent false conclusions from being drawn (e.g. on the existence of bias), implying that accurate assessments of data quality require choosing the right statistical tools. Finally, we consider repeat survey data to be indispensable because they provide an independent measure of uncertainty, which is of critical importance when assessing biodiversity changes in times of ongoing global change.

## Supplementary information

**Additional file 1.** Supplementary material on methods and results.

## Data Availability

The datasets used and/or analysed during the current study are available from the corresponding author on reasonable request.

## Abbreviations

CI: Confidence interval
CL: Confidence limit
CV: Coefficient of variation
DQO: Data quality objectives
DQR: Data quality results
ICP Forests: International Co-operative Programme on Assessment and Monitoring of Air Pollution Effects on Forests
med: median
MQO: Measurement quality objectives
NFI: National Forest Inventory
PT: Pseudo-turnover of species composition
QA: Quality assessment
stddev: Standard deviation
stderr: Standard error
TOST: Two one- sided t-tests
