# Supplementary material for: Analysing the quality of Swiss National Forest Inventory measurements of woody species richness
Source: For Ecosyst. 2020 Jun 17;7(1):37. doi: 10.1186/s40663-020-00252-1 (PMC7357775; doi:10.1186/s40663-020-00252-1)
Supplement: Supplementary file 1 — Additional file 1. Supplementary material on methods and results. [file 40663_2020_252_MOESM1_ESM.docx]

# Additional File 1: Supplementary material on methods and results

## Observer bias

According to Lakens (2017), the t-values for the two one-sided tests for a one-sample t-test are:

| $t_{L=}\frac{M-\mu-\Delta_{L}}{\frac{SD}{\sqrt{N}}} \mathrm{and} t_{U=}\frac{M-\mu-\Delta_{U}}{\frac{SD}{\sqrt{N}}}$ | Eq. S1 |
| --- | --- |

where *M* is the observed mean, *SD* is the observed standard deviation, *N* is the sample size and $\mu$ is the value that the mean is tested against. The upper and lower equivalence bounds of practical relevance are denoted as $\Delta_{L}$ and $\Delta_{U}$; they correspond to the critical margins $m$ as used in the main part of the study.

The interpretation of TOST-based confidence intervals can be derived from Fig. S1. They show mean differences (dots) and 90% (($1-2\alpha$) × 100%) confidence intervals (horizontal lines) with equivalence bounds $\Delta_{L}$ = –1 and $\Delta_{U}$= 1 as an example for several combinations of test results. The reason for the ($1-2\alpha$) × 100% CI is that the TOST method is based on the concurrent rejection of two one-sided tests at the level of $1-\alpha$. Thus the 90% CI yields an $\alpha=0.05$ significance level for testing equivalence. Type A in Fig. S1 is statistically equivalent but not different, and the sample size is sufficiently large to detect equivalence; type B is statistically different from 0 and not equivalent; type C is practically insignificant, i.e. there is a difference but it is trivial; type D is inconclusive, i.e. it is neither statistically different from 0 nor equivalent, and the variability is too great relative to the effect size to interpret (the study might be underpowered); and type E is inconclusive as well, with the conjecture probably true but the data too variable to be certain.


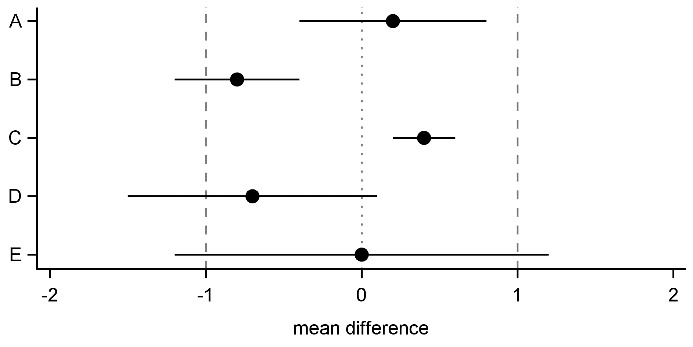


**Figure S1** Interpretation of TOST CI (adapted from Lakens 2017). Dots: mean richness differences, horizontal lines: 90% confidence intervals, dashed lines: equivalence bounds $\Delta_{L}$ = –1 and $\Delta_{U}$= 1 as an example.

Power analysis for equivalence tests


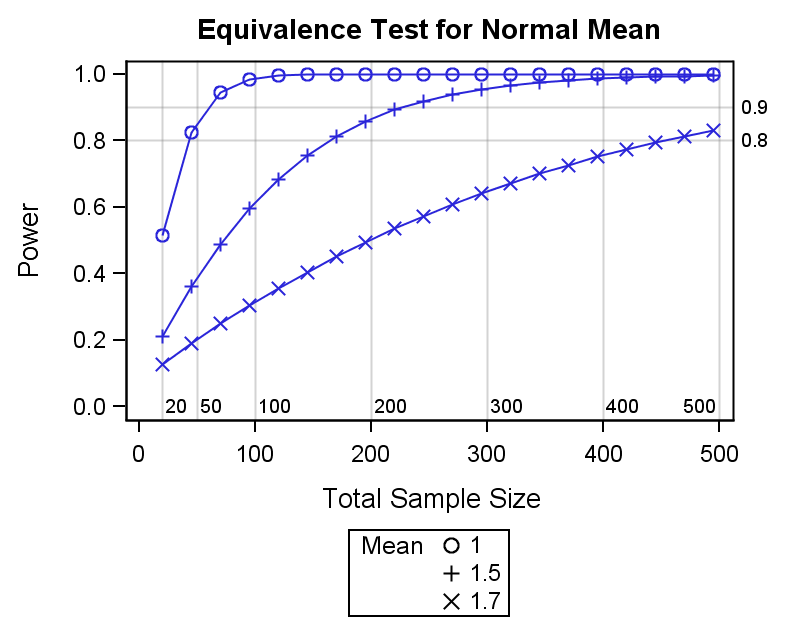


**Figure S2** Power and sample size for the equivalence test of woody species (WoodySp). TOST margins: ±2, alpha = 0.05, stddev = 2.56 (derived from NFI4 richness differences). The symbols of the mean represent the effect sizes, which are the expected mean absolute richness differences (distance from zero).

## DQO analysis

**Table S1** DQR statistics including Wilson lower and upper confidence limits. The DQR values represent the percentage of cases where the MQO threshold has been reached. Database: repeat survey of NFI3 and NFI4.

| Attribute | NFI | MQO | Mean | DQR | DQR | | *n* | Nested (%) |
| --- | --- | --- | --- | --- | --- | --- | --- | --- |
|  |  | (b + c) | (b + c) | (%) | LowerCL (%) | UpperCL (%) |  |  |
| WoodySp | 4 | 2 | 3.74 | 50.46 | 45.79 | 55.11 | 438 | 34.47 |
|  | 3 | 2 | 3.82 | 50.16 | 46.25 | 54.06 | 626 | 31.79 |
| FoEdge | 4 | 3 | 4.48 | 50.00 | 37.74 | 62.26 | 60 | 33.33 |
|  | 3 | 3 | 4.27 | 46.32 | 36.63 | 56.29 | 95 | 38.95 |
| UpStorey | 4 | 0 | 0.61 | 64.93 | 59.04 | 70.39 | 268 | 24.25 |
|  | 3 | 0 | 0.86 | 51.51 | 46.61 | 56.38 | 398 | 39.20 |

Legend: MQO: measurement quality objectives (the maximum accepted number of exclusive species (b + c) where b is number of species reported only in the regular survey and c is the number of species reported only in the repeat survey, Mean: the arithmetic mean of (b + c), DQR: data quality results, LowerCL: lower confidence limit, UpperCL: upper confidence limit, Nested: percentage of nested sample plots (the species composition of one survey is a subset of the composition of the other survey).


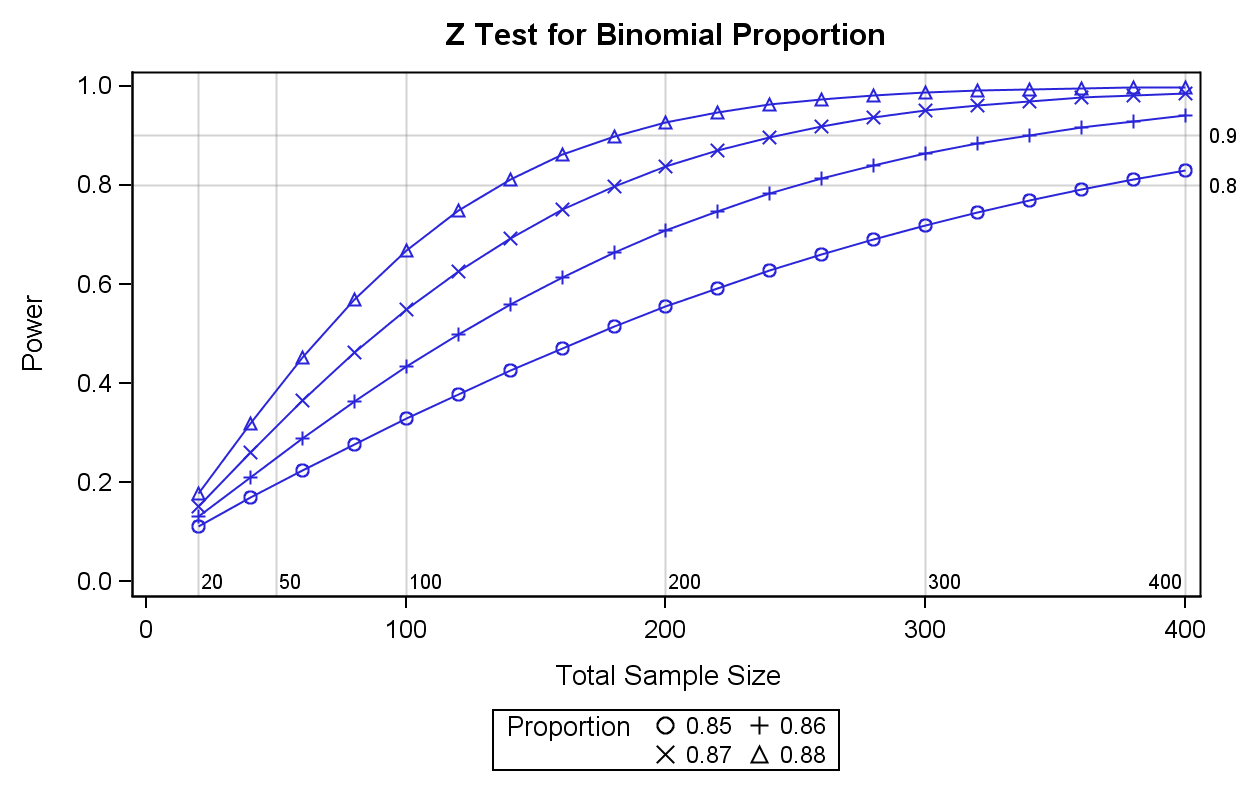


**Figure S3** Results from the power analysis for the binomial z-tests (normal approximation to the binomial distribution). $H_{0}=0.8$, proportion: assumed data quality results (DQR).

## PT analysis

Both the numerator and denominator of the PT values are interpreted as random variables, and thus a ‘ratio of means’ estimator according to Cochran (1977) (eqs. 2.47 / 6.13) was calculated to derive the mean and standard error for CI construction. We compared the ‘ratio of means’ results to the means and CIs of arcsine and logit transformed data, as recommended by Zar (2010), and also to estimators directly calculated from the PT values of the sample plots (‘mean of ratio’ method). From the overlapping CIs in Figure S4, we concluded that all methods, except the arcsine transformation for the attribute UpStorey, yield comparable results.


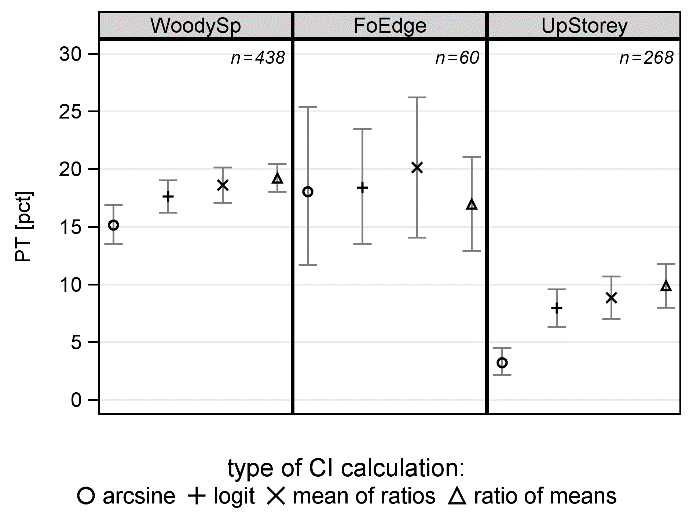


**Figure S4** Comparison of several methods to calculate PT mean values and confidence intervals derived from NFI4 data.

**Table S2** Results from the PT analysis. Statistics are derived from the ratio of means method, all values except n are percent values. Data basis: repeat survey of NFI3 and NFI4.

| Attribute | NFI | Mean | Median | Stddev | Stderr | LowerCL | UpperCL | *n* |
| --- | --- | --- | --- | --- | --- | --- | --- | --- |
| WoodySp | 4 | 19.22 | 15.79 | 12.64 | 0.60 | 18.03 | 20.41 | 438 |
|  | 3 | 22.98 | 20.00 | 15.18 | 0.61 | 21.79 | 24.17 | 626 |
| FoEdge | 4 | 16.95 | 15.39 | 15.73 | 2.03 | 12.89 | 21.01 | 60 |
|  | 3 | 18.61 | 17.24 | 15.60 | 1.60 | 15.43 | 21.79 | 95 |
| UpStorey | 4 | 9.88 | 0.00 | 15.81 | 0.97 | 7.98 | 11.78 | 268 |
|  | 3 | 15.45 | 0.00 | 18.97 | 0.95 | 13.58 | 17.32 | 398 |

Legend: WoodySp: woody species, FoEdge: forest edge species, UpStorey: upper storey species, stddev: standard deviation, stderr: standard error, LowerCL: lower 95% confidence limit, UpperCL: upper 95% confidence limit.

**Table S3** Statistics of the components of PT. All values represent the number of species. Data basis: repeat survey of NFI3 and NFI4.

| Attribute | NFI | Mean | |  | Med | |  | Stddev | |  | Stderr | |
| --- | --- | --- | --- | --- | --- | --- | --- | --- | --- | --- | --- | --- |
|  |  | a | b+c |  | a | b+c |  | a | b+c |  | a | b+c |
| WoodySp | 4 | 7.86 | 3.74 |  | 6 | 2.00 |  | 5.61 | 3.76 |  | 0.27 | 0.18 |
|  | 3 | 6.40 | 3.82 |  | 6 | 2.00 |  | 4.40 | 3.83 |  | 0.18 | 0.15 |
| FoEdge | 4 | 10.98 | 4.48 |  | 11 | 3.50 |  | 5.04 | 4.00 |  | 0.65 | 0.52 |
|  | 3 | 9.35 | 4.27 |  | 10 | 4.00 |  | 5.13 | 3.23 |  | 0.53 | 0.33 |
| UpStorey | 4 | 2.79 | 0.61 |  | 3 | 0.00 |  | 1.46 | 1.06 |  | 0.09 | 0.06 |
|  | 3 | 2.36 | 0.86 |  | 2 | 0.00 |  | 1.23 | 1.17 |  | 0.06 | 0.06 |

Legend: WoodySp: woody species, FoEdge: forest edge species, UpStorey: upper storey species, a: species found in both surveys, b + c: sum of exclusive species, stddev: standard deviation, stderr: standard error.

Power analysis


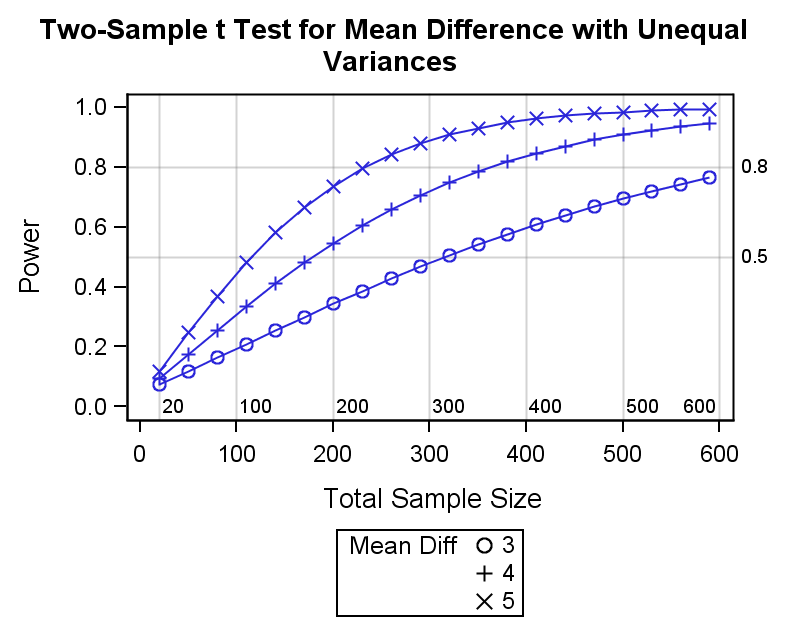


**Figure S5** Power analysis based on the two-sample means t-test for unequal variances for the attribute woody species (WoodySp). Parameters: stddev NFI3 = 15, stddev NFI4 = 12, sample weight NFI3 to NFI4 = 3/2, $\alpha=0.05$. Mean Diff: Difference in PT in % (effect), stddev: standard deviation.
